# An easy route to the massive karyotyping of complex chromosomal arrangements in Drosophila

**DOI:** 10.1038/s41598-017-13043-6

**Published:** 2017-10-05

**Authors:** Dorcas J. Orengo, Eva Puerma, Unai Cereijo, David Salguero, Montserrat Aguadé

**Affiliations:** Departament de Genètica, Microbiologia i Estadística, Facultat de Biologia and Institut de Recerca de la Biodiversitat (IRBio), Universitat de Barcelona, Diagonal 643, 08028 Barcelona, Spain

## Abstract

Inversion polymorphism is widespread in the Drosophila genus as well as in other dipteran genera. The presence of polytene chromosomes in some insect organs and, thus, the possibility to observe the different arrangements generated by inversions through a microscope enhanced the cytological study of this structural polymorphism. In several Drosophila species, these studies provided evidence for the adaptive character of this polymorphism, which together with the standing interest to uncover targets of natural selection has led to a renewed interest for inversion polymorphism. Our recent molecular characterization of the breakpoint regions of five inversions of the E chromosome of *D*. *subobscura* has allowed us to design a PCR-based strategy to molecularly identify the different chromosomal arrangements and, most importantly, to determine the E chromosome karyotype of medium- and large-sized samples from natural populations. Individuals of a test sample that were both cytologically and molecularly karyotyped were used to establish the strategy that was subsequently applied to karyotype a larger sample. Our strategy has proved to be robust and time efficient, and it lays therefore the groundwork for future studies of the E chromosome structural polymorphism through space and time, and of its putative contribution to adaptation.

## Introduction

Classical cytological studies of structural variation in the Drosophila genus revealed the widespread character of paracentric inversion polymorphism in this genus. Its geographical distribution has been extensively studied in such diverse species as *D*. *melanogaster*, *D*. *pseudoobscura*, *D*. *subobscura* and *D*. *buzzatii* ^1^, where it has been considered to be adaptive and thus maintained by natural selection. The more restricted temporal studies performed in some of these species allowed the detection of some instances of seasonal variation (*e*. *g*., in *D*. *pseudoobscura* and *D*. *subobscura* ^2,3^). Temporal studies also revealed the generally stable character of chromosomal polymorphism across multiple decades (*e*. *g*., in *D*. *pseudoobscura* and *D*. *subobscura* ^4–6^) despite the detection in some cases of subtle changes through time concordant with changes in environmental variables (*e*. *g*., in *D*. *subobscura* ^6–9^). Although the strongest support for Drosophila chromosomal polymorphism adaptive character stems from the observation in both *D*. *melanogaster* and *D*. *subobscura* of parallel latitudinal clines in different continents^10–12^, the seasonal and temporal changes detected in *D*. *pseudoobscura* and *D*. *subobscura* and their relationship with environmental changes also point to positive selection as the underlying mechanism for the spread and establishment of inversions.

**Figure 1:**
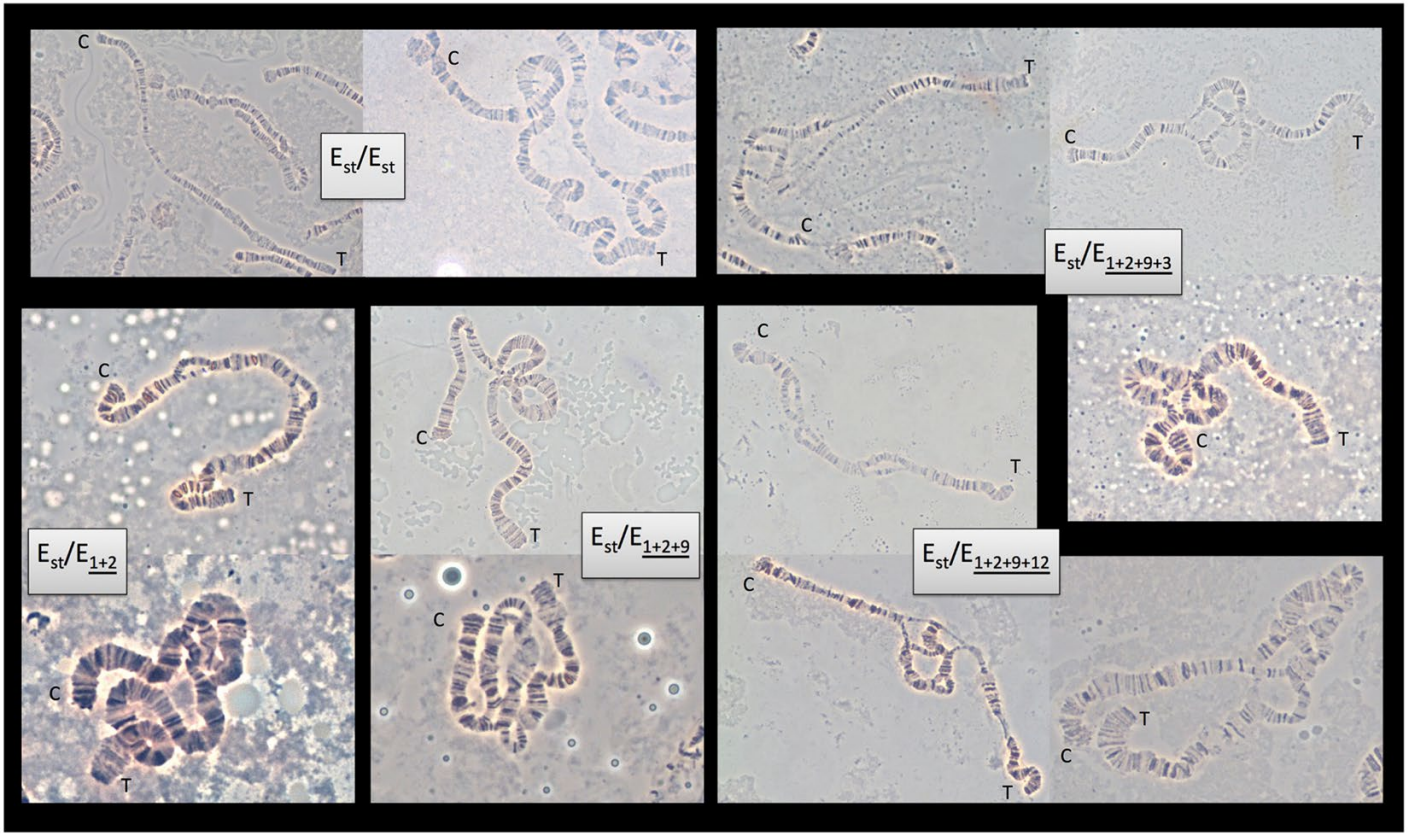
Micrographs of polytene chromosomes corresponding to homokaryotypic and heterokaryotypic individuals for E chromosome arrangements.

The cytological characterization of chromosomal polymorphism in natural populations is a laborious endeavor even if performed in larvae obtained after crossing wild-caught males with virgin females from a laboratory strain homokaryotypic for all chromosomal arms. Although the frequencies of the different chromosomal arrangements that segregate in a population can be estimated through the observation of a single polytene chromosomes preparation per wild caught male, at least seven preparations from F_1_ larvae need to be observed to estimate global karyotype frequencies with a certain probability. Moreover, the considerable expertise required to reliably identify the different inversions that might be carried by an individual constitutes an additional difficulty and possibly limiting step in this procedure, especially when complex chromosomal arrangements such as those of the E chromosome of *D*. *subobscura* are considered (Fig. 1).

The molecular characterization of inversion breakpoints in inverted and non-inverted chromosomes allows the development of PCR-based assays to detect the corresponding inversion presence or absence in any individual, and therefore to establish its karyotype. Such assays have been designed for seven inversions that segregate in *D*. *melanogaster* natural populations^13–16^, even though these assays await their use to estimate inversion frequencies in natural populations.

The chromosomal polymorphism of *D*. *subobscura* stands out because it affects the species five large acrocentric chromosomes even though inversions are not equally numerous across chromosomal elements. It also stands out because some of its inversions form complex arrangements that can adopt more than one configuration when present in heterozygosis (Fig. 1). The E chromosome (Muller’s C element) is among those that exhibit complex arrangements resulting from partially overlapping and sequentially originated inversions. The recent characterization of the breakpoints of five of these inversions —E_1_, E_2_, E_9_, E_3_ and E_12_— in inverted and non-inverted chromosomes^17–19^ has prompted us to design a robust and efficient PCR-based strategy to molecularly karyotype medium- to large-sized samples from natural populations, which will allow the characterization of the E chromosome structural polymorphism both at the geographical and temporal levels, and therefore the detection of putative changes not only of arrangements frequencies but also of karyotypes frequencies and their relationship with diverse environmental and biotic variables.

## Results

### Cytology-based karyotyping of wild-caught males

**Figure 2:**
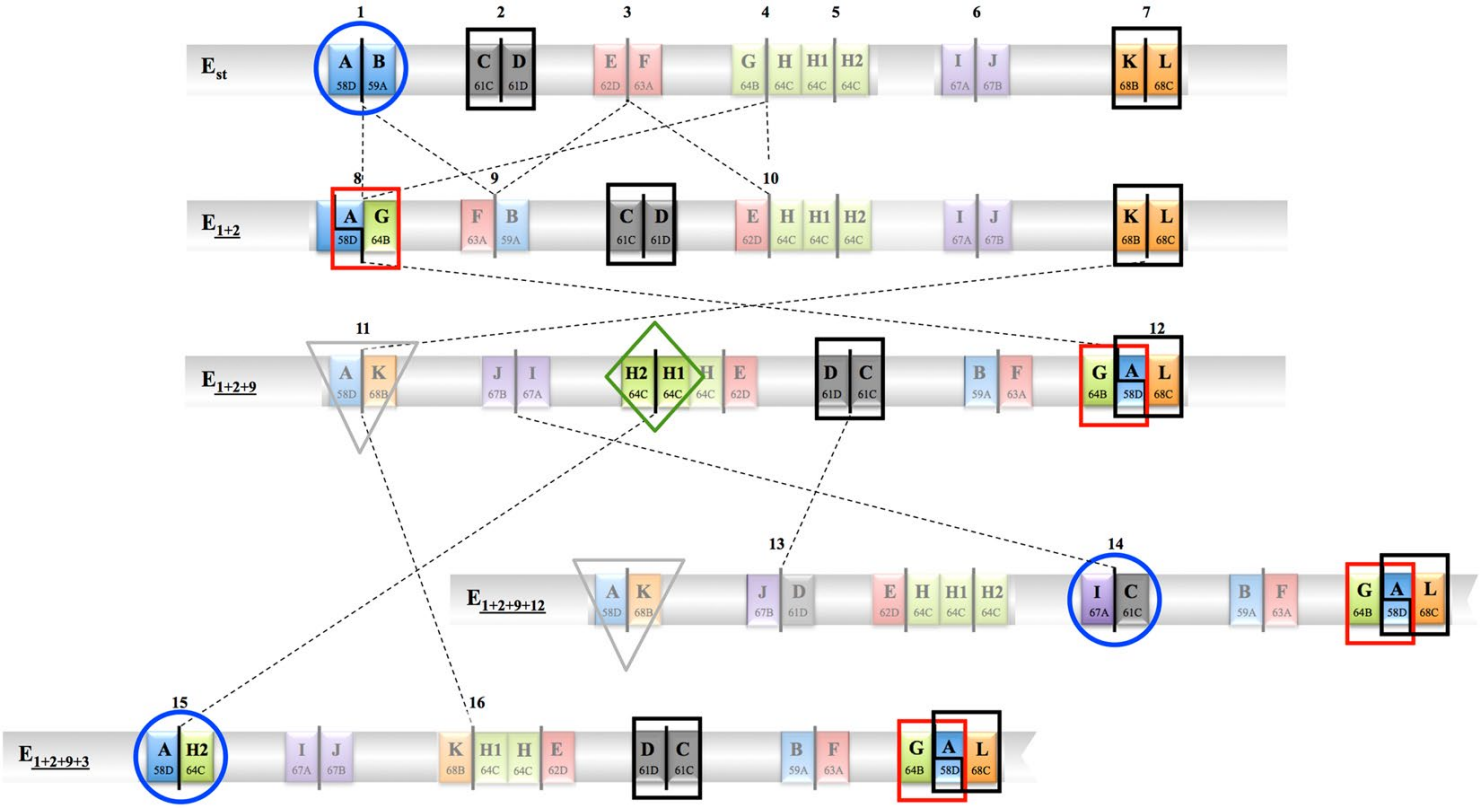
Schematic representation of the five E chromosome arrangements considered in this study and their breakpoint regions. Discontinuous lines connecting two chromosomal arrangements indicate the inversions that differentiate them. On each chromosomal arrangement, the breakpoints involved in inversions E_1_, E_2_, E_9_, E_3_ and E_12_ are indicated, although only those used for karyotyping are highlighted. Circles indicate fragments exclusive of one arrangement (fully diagnostic). Rectangles indicate fragments that are shared by two or more arrangements. Inverted triangles indicate an initially used fragment (AK) that later was discarded due to its high failure to amplify frequency. A rhombus indicates a fragment that is only used to discern between the E_1+2+9+3_/E_1+2+9+3_ and E_1+2+9_/E_1+2+9+3_ karyotypes.

Twenty-five males collected at Observatori Fabra in November 2014 were used to establish a strategy to molecularly determine the karyotypes involving the five most frequent E chromosomal arrangements —E_st_, E_1+2_, E_1+2+9_, E_1+2+9+3_ and E_1+2+9+12_ (Fig. 2)— in the sampled population as well as in other western European populations^5,6,20,21^. Individuals of this test set were first cytologically karyotyped in order to later validate the molecular karyotyping strategy. Table 1 shows the karyotypes determined for 24 of these males and the only E chromosome arrangement that could be assigned to individual 21 M. All five chromosomal arrangements were present in the sampled males and ten of the fifteen possible karyotypes were detected (Table 1).

**Table 1:** PCR amplification results for 25 cytologically karyotyped males.

♂♂	Cytological Karyotype		PCR fragments^1^									Molecular Karyotype	
			AB	AH2	I C	AK	AG	KL	CD	AL	H1H2		
1 M	E_st_	E_st_	+					+	+		+	E_st_	E_st_
2 M	E_st_	E_st_	+					+	+		+	E_st_	E_st_
3 M	E_1+2_	E_1+2+9+12_			+	+	+ +	+	+	+	+	E_1+2_	E_1+2+9+12_
4 M	E_st_	E_1+2_	+				+	+	+		+	E_st_	E_1+2_
5 M	E_st_	E_1+2+9+12_	+		+	+	+	+	+	+	+ +	E_st_	E_1+2+9+12_
6 M	E_1+2+9_	E_1+2+9+12_			+	+	+		+	+	+	E_1+2+9_	E_1+2+9+12_
7 M	E_st_	E_1+2_	+				+	+	+		+	E_st_	E_1+2_
8 M	E_st_	E_st_	+					+	+		+	E_st_	E_st_
9 M	E_1+2_	E_1+2+9+3_		+			+	+	+	+	+	E_1+2_	E_1+2+9+3_
10 M	E_st_	E_st_	+					+	+		+	E_st_	E_st_
11 M	E_st_	E_st_	+					+	+		+	E_st_	E_st_
12 M	E_1+2+9+12_	E_1+2+9+12_			+ +	+	+			+	+	E_1+2+9+12_	E_1+2+9+12_
13 M	E_1+2_	E_1+2+9+12_			+	+	+	+	+	+	+	E_1+2_	E_1+2+9+12_
14 M	E_st_	E_1+2_	+				+	+	+		+	E_st_	E_1+2_
15 M	E_1+2+9_	E_1+2+9+12_			+	+	+		+	+ +	+	E_1+2+9_	E_1+2+9+12_
16 M	E_st_	E_1+2+9+12_	+		+	+	+	+	+	+	+	E_st_	E_1+2+9+12_
17 M	E_st_	E_1+2+9+3_	+	+			+	+	+	+	+	E_st_	E_1+2+9+3_
18 M	E_1+2_	E_1+2+9_	+			+	+	+	+	+	+	E_1+2_	E_1+2+9_
19 M	E_st_	E_1+2+9_	+		+	+	+	+	+	+	+	E_st_	E_1+2+9_
20 M	E_st_	E_1+2+9+12_			+	+	+	+	+	+	+	E_st_	E_1+2+9+12_
21 M	E_1+2+9+12_	??			+	+	+	+	+	+	+	E_1+2_	E_1+2+9+12_
22 M	E_1+2+9+12_	E_1+2+9+12_	+				+			++	+	E_1+2+9+12_	E_1+2+9+12_
23 M	E_st_	E_1+2+9_	+				+	+	+	+	+	E_st_	E_1+2+9_
24 M	E_st_	E_1+2+9_	+				+	+	+	+	+	E_st_	E_1+2+9_
25 M	E_st_	E_1+2_					+	+	+		+	E_st_	E_1+2_

The karyotype of 24 of the 25 males collected in November 2014 could be cytologically established whereas for male 21 M, only one of its E chromosome arrangements could be identified. Fragments AB, AH2 and IC are exclusive for arrangements E_st_, E_1+2+9+3_ and E_1+2+9+12_, respectively. +, ﻿a single amplification product; ++, two differently sized amplification products.

^1^Initally, the amplification of seven PCR fragments (AB, AH2, IC, AK, AG, KL and CD) was tested in the 25 males sample. The relatively high dropout frequency of fragment AK detected in the 2015 sample led to its replacement by fragment AL, which required the additional amplification of fragment H1H2 to discern between the low-frequency karyotypes E_1+2+9_/E_1+2+9+3_ and E_1+2+9+3_/E_1+2+9+3_ (see text).

### Establishing the molecular karyotyping strategy

The complexity of the system here studied and its effect on the molecular karyotyping of wild-caught individuals is highlighted in Fig. 2. This complexity stems from four different aspects: i) the reuse of the most proximal breakpoint by inversions E_1_ (or E_2_), E_9_ and E_3_^17,18^; ii) the extinction of the intermediate arrangement leading from E_st_ to E_1+2_; iii) the sequential occurrence of inversions E_9_, E_3_ and E_12_ leading from E_1+2_ to E_1+2+9_, and from E_1+2+9_ to both E_1+2+9+3_ and E_1+2+9+12_; and iv) the duplications generated by the process originating several of these inversions. The first two aspects (reuse of one breakpoint by three inversions and absence of an intermediate arrangement) imply that when breakpoint regions in each extant ancestral and derived chromosomal arrangement are considered, 16 (instead of 20) have been affected (Fig. 2 and Supplementary Fig. S1). Moreover, the sequential occurrence of inversions (third aspect) implies that some of the breakpoint regions resulting from the origin of the first arrangement are also present in the subsequent arrangements (*i*. *e*., fragments FB and EH in E_1+2_, E_1+2+9_, E_1+2+9+3_ and E_1+2+9+12_, fragment GAL in E_1+2+9_, E_1+2+9+3_ and E_1+2+9+12_, and fragment AK in E_1+2+9_ and E_1+2+9+12_; Fig. 2). Finally, the duplication generated by the origin of an inversion (fourth aspect) implies that the duplicated region is present not only in the first derived arrangement but also in subsequent arrangements. When the duplicated region is large (as is the case of the ~7.8-kb long A region^18^), this might preclude the differential amplification of the breakpoint region in the ancestral (AG fragment in E_1+2_) and derived (GAL fragment in E_1+2+9_) arrangements (Fig. 2).

**Figure 3:**
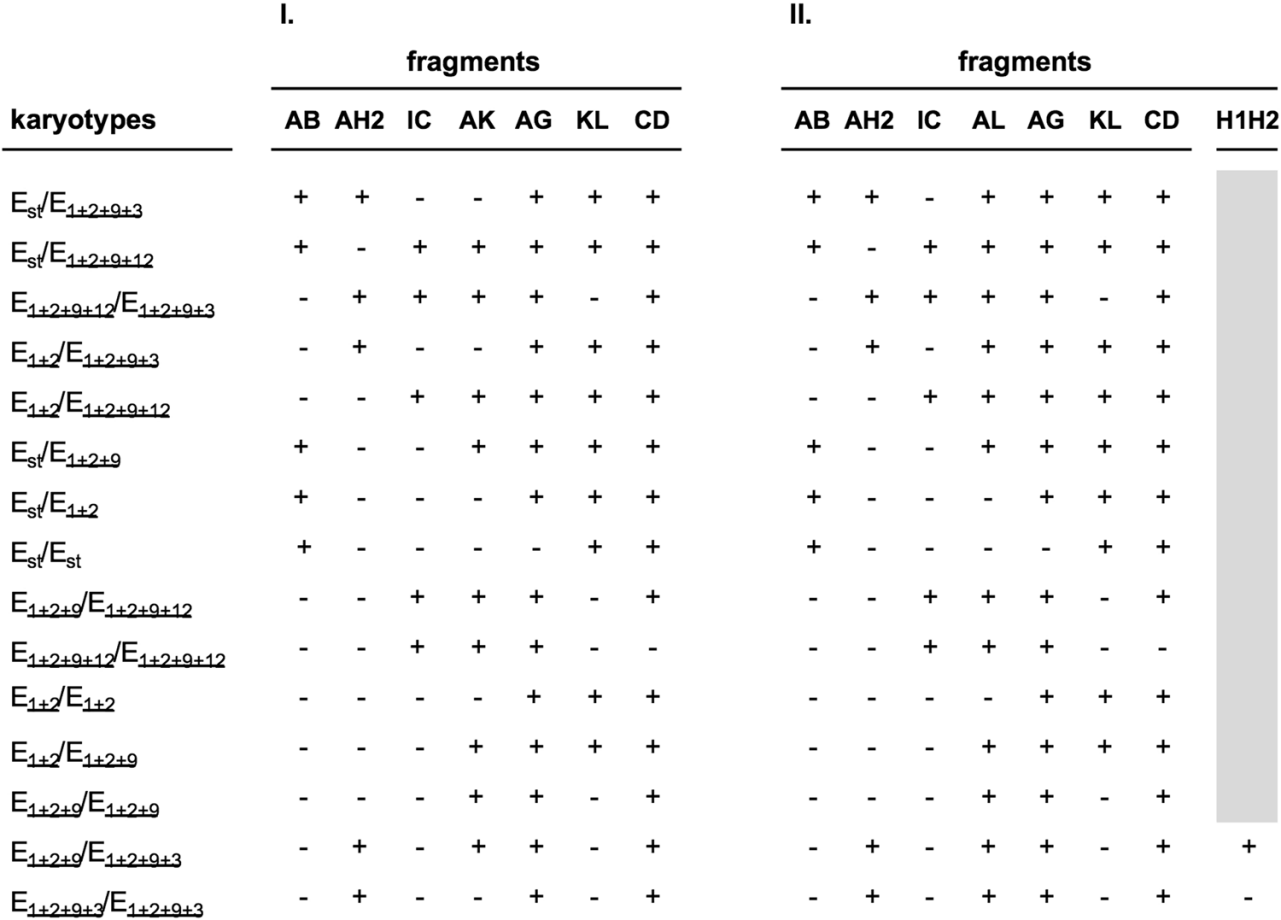
Strategy used to determine each of the 15 possible karyotypes for chromosomal arrangements E_st_, E_1+2_, E_1+2+9_, E_1+2+9+3_ and E_1+2+9+12_. (I) Initial one-round strategy consisting in the PCR amplification of seven fragments. (II) Final two-rounds strategy consisting in a first round of seven PCR amplifications, and a second conditional round of a single amplification. + and −, expected and not expected fragment amplification, respectively.

Supplementary Fig. S1 shows which of 16 fragments are expected to amplify in individuals that carry each of the five E chromosomal arrangements here considered. This figure clearly shows that there are pairs (and triplets) of fragments which amplification provides the same information and that only chromosomal arrangements E_st_, E_1+2+9+3_ and E_1+2+9+12_ exhibit exclusive breakpoint regions (AB, EF and GH for E_st_; AH2 and KH1 for E_1+2+9+3_; JD and IC for E_1+2+9+12_; Fig. 2 and Supplementary Fig. S1). Accordingly, results of the PCR reactions of seven fragments —AB (or either EF or GH), AG (or either FB or EH), AK, AH2 (or KH1), JD (or IC), KL and CD (or IJ)— could be sufficient to identify the fifteen possible karyotypes for the five chromosomal arrangements (Fig. 3).

The extent of each fragment to be amplified for karyotyping purposes and the initial set of primers used to PCR amplify at least one of each of the seven above-mentioned groups of diagnostic fragments were based on sequences obtained during the initial identification of inversions E_1_, E_2_, E_9_, E_3_ and E_12_ breakpoint regions^17–19^. Amplification efficiency was initially tested in homokaryotypic lines. It was subsequently tested in heterozygosis, firstly by generating DNA from mocked heterokaryotypic individuals by pairwise combining DNAs from homokaryotypic lines with different chromosomal arrangements in different proportions (1:3, 1:1, 3:1), and secondly by using heterokaryotypic individuals obtained from crosses between homokaryotypic lines. All fragments did successfully amplify in homokaryotypic and heterokaryotypic flies as well as in the different mocked heterokaryotypic conditions generated.

The molecular strategy using seven fragments —AB, AG, AK, AH2, JD, KL and CD (Supplementary Fig. S1)— was applied to the 24 males with cytologically determined karyotypes. The PCR-based karyotypes reproduced in most cases the cytological results. For those primer pairs and fragments that according to the male cytology-based karyotype were expected to amplify (Supplementary Fig. S1) and had failed to do so in at least one of the males, different strategies were used to obtain robust primer pairs and amplification conditions. These strategies included i) assaying new annealing and extension temperatures, and ii) designing primer pairs to amplify alternative diagnostic fragments (*e*. *g*., fragment IC as opposed to fragment JD; Fig. 2). The presence of inversion E_12_ was particularly difficult to be consistently established, most possibly due to nucleotide and length polymorphism at its breakpoint regions. Indeed, the JD fragment failed to amplify in three of the nine males with at least one E_1+2+9+12_ arrangement even after assaying several primer pairs and PCR conditions. The consistent amplification of the E_12_ alternative diagnostic fragment (IC) also required assaying several primer pairs and PCR conditions, but a final primer pair did successfully identify its presence in all cases. The final set of fragments used to establish the molecular karyotyping strategy (*i*. *e*., through the PCR amplification of seven fragments in the test set of 24 males collected in 2014) is that depicted in Table 1. This table shows the amplification results not only for these 24 males but also for male 21 M, which allowed the establishment of its karyotype.

### Testing the molecular karyotyping strategy in random samples

The strategy was later applied to two sets of individuals with unknown karyotype that had been also collected at Observatori Fabra: 25 females in November 2014, and 96 individuals (48 males and 48 females) in November 2015. Although the same seven fragments were amplified in both sets, amplification reactions were performed in individual tubes in the first set —as done in our test set of males— and in 96-well plates using multi-channel micropipettes in the second set, which would allow us to check the robustness of our strategy using this higher throughput procedure. Supplementary Tables S1 and S2 present the amplification results for the 25 females and 96 individuals, respectively, whereas Supplementary Fig. S2 shows the electrophoresis images corresponding to 12 of the 48 males collected in November 2015 and the inferred E chromosome karyotypes.

The successful amplification of one fragment implies in some cases that one or more additional fragments should also amplify (*e*. *g*., in individuals where fragment AH2 has amplified, fragments AG and CD are also expected to do so; Supplementary Fig. S1). It is important to highlight that these conditional amplifications allowed us to evaluate the robustness of our molecular karyotyping strategy in the 121 individuals that had not been cytologically karyotyped. Failure to amplify amounted to 1 out of 74 KL fragments, 0 out of 93 CD fragments, 3 out of 80 AG fragments and 7 out of 61 AK fragments.

The primer pairs and PCR conditions established for fragment AK using the test set of 25 males proofed to be the least robust. As previously indicated, the large size of the A duplication present in the GAL fragment precluded us to switch from the AK to the GAL fragment. We therefore designed primers to amplify the AL fragment that was expected to amplify in all individuals carrying at least one copy of chromosomal arrangements E_1+2+9_, E_1+2+9+3_ and E_1+2+9+12_ (*i*. *e*., in all individuals where the AK fragment was expected to amplify plus in individuals carrying at least one copy of the E_1+2+9+3_ chromosomal arrangement; Fig. 2 and Supplementary Fig. S1). One of the designed primer pairs and set of PCR conditions assayed did successfully amplify the AL fragment in all the cytologically karyotyped males where it was expected to do so (16) and in none where it was not (9; Table 1). The successful primer pair and PCR conditions were then used to amplify the AL fragment in the 25 females collected in 2014 and in the 96 individuals collected in 2015.

In these samples, fragment AL amplified in 19 and 61 individuals, respectively, implying the presence of the E_9_ inversion. In this way, the karyotype of 24 of the 25 females collected in 2014, and of 93 of the 96 individuals collected in 2015 could be assigned according to the amplification results of fragments AB, AH2, IC, AL, AG, KL and CD (Supplementary Tables S2 and S3). However, because arrangements E_1+2+9,_ E_1+2+9+3_ and E_1+2+9+12_ share the AL breakpoint region, information from the H1H2 fragment was required for the E_1+2+9_/E_1+2+9+3_ and E_1+2+9+3_/E_1+2+9+3_ karyotypes discrimination in the 4 remaining individuals (Figs 2 and 3). The final set of primer pairs and amplification conditions for the different fragments is given in Supplementary Table S3.

### Analysis of karyotype and chromosomal arrangement frequencies

Table 2 gives the frequencies estimated in the present study of the fifteen possible karyotypes for chromosomal arrangements E_st_, E_1+2_, E_1+2+9_, E_1+2+9+3_ and E_1+2+9+12_ in individuals collected at Observatori Fabra in November 2014 and 2015, whereas Table 3 gives those of the five chromosomal arrangements. Thirteen and 14 of the 15 possible karyotypes were detected in the 2014 and 2015 samples, respectively. A simple inspection of the karyotype frequencies revealed that they were rather similar between sexes in the 2014 sample but not in the 2015 sample (Table 2). When the frequencies of the four E chromosome arrangements with absolute frequencies ≥ 5 (Table 3) were compared between sexes, they did not differ significantly in either case even though frequency differences were marginally significant in the 2015 sample (χ^2^ = 3.986; d.f. = 3; P = 0.263; χ^2^ = 6.594; d.f. = 3; P = 0.086, in the 2014 and 2015 samples, respectively). It should be noted that in the latter sample, the major contribution to the χ^2^ value was that of the E_1+2+9+12_ arrangement (4.66), reflecting its lower frequency in females.

**Table 2:** Karyotype frequencies in samples from two consecutive years.

Karyotypes	2014 sample			2015 sample			Total			
	♂♂	♀♀	Total	♂♂	♀♀	Total	♂♂	♀♀	Total	%
E_st_/E_st_	5	3	8	8	10	18	13	13	26	17.81
E_st_/E_1+2_	4	3	7	6	9	15	10	12	22	15.07
E_st_/E_1+2+9_	3	4	7	4	6	10	7	10	17	11.64
E_st_/E_1+2+9+3_	1	0	1	1	0	1	2	0	2	1.37
E_st_/E_1+2+9+12_	3	5	8	9	6	15	12	11	23	15.75
E_1+2_/E_1+2_	0	0	0	0	2	2	0	2	2	1.37
E_1+2_/E_1+2+9_	1	1	2	2	4	6	3	5	8	5.48
E_1+2_/E_1+2+9+3_	1	1	2	0	1	1	1	2	3	2.05
E_1+2_/E_1+2+9+12_	3	0	3	5	2	7	8	2	10	6.85
E_1+2+9_/E_1+2+9_	0	3	3	1	2	3	1	5	6	4.11
E_1+2+9_/E_1+2+9+3_	0	1	1	2	1	3	2	2	4	2.74
E_1+2+9_/E_1+2+9+12_	2	1	3	6	3	9	8	4	12	8.22
E_1+2+9+3_/E_1+2+9+3_	0	0	0	0	0	0	0	0	0	0.00
E_1+2+9+3_/E_1+2+9+12_	0	1	1	0	1	1	0	2	2	1.37
E_1+2+9+12_/E_1+2+9+12_	2	2	4	4	1	5	6	3	9	6.16
Total	25	25	50	48	48	96	73	73	146	100.00

**Table 3:** Arrangement frequencies in samples from two consecutive years.

Arrangements	2014 sample			2015 sample			Total			
	♂♂	♀♀	Total	♂♂	♀♀	Total	♂♂	♀♀	Total	%
E_st_	21	18	39	36	41	77	57	59	116	39.73
E_1+2_	9	5	14	13	20	33	22	25	47	16.10
E_1+2+9_	6	13	19	16	18	34	22	31	53	18.15
E_1+2+9+3_	2	3	5	3	3	6	5	6	11	3.77
E_1+2+9+12_	12	11	23	28	14	42	40	25	65	22.26
Total	50	50	100	96	96	192	146	146	292	100.00

## Discussion

In this study, we developed a robust and efficient strategy to molecularly identify the most frequent E chromosome arrangements in western European populations of *D*. *subobscura*, as well as to determine the karyotype of wild-caught individuals of either sex. Our strategy to molecularly karyotype individuals through the PCR amplification of fragments including inversion breakpoints offers several advantages over their karyotyping through cytological methods. The most obvious advantages are i) the reduced time needed for the fast screening of large samples and ii) the reduced expertise required to perform the diagnostic experiments as opposed to that required for the correct identification of complex chromosomal arrangements —such as those of the E chromosome— even if in heterozygosis over a standard chromosome. An additional and important advantage of any molecular karyotyping strategy is that it can be directly applied to both wild-caught males and females unlike cytology-based karyotyping methods that require the observation of polytene chromosomes of third-instar larvae after crossing wild-caught flies with a reference strain. This developmental stage limitation implies that only the karyotype of wild-caught males can be determined by cytological methods because controlled crosses cannot be performed with gravid females.

Our previous characterization of the breakpoints of the five E chromosome inversions here studied revealed the presence of transposable elements, or remnants thereof, in most of the inverted breakpoint regions^17–19^. This characterization also revealed that in some cases other structural rearrangements (*e*. *g*., microinversions and small insertions/deletions) had occurred in these regions or in their vicinity^17–19^. These characteristics have affected our efforts to establish a robust and time-efficient strategy to molecularly karyotype high numbers of wild-caught individuals of either sex through the PCR amplification of diagnostic fragments. Our test set consisted in 25 males that we first karyotyped by cytological methods. Designing primer pairs and establishing PCR conditions of the different fragments that allowed the correct assignment of each male karyotype was not an easy endeavor. As previously explained, the successful amplification of a fragment exclusive of a particular arrangement implies that other fragments should also amplify (*e*.* g*., fragment KL if fragment AB had amplified). This conditional expectation for certain fragments amplification allowed us to detect some failures to amplify. Dropout frequency was low in three of the four initial fragments not exclusive of a particular arrangement —AG, KL and CD—, but relatively high in the fourth fragment (AK). After switching our strategy from a one-round of seven amplifications (including the AK fragment) to a conditional two-rounds of seven and one amplifications, respectively, the dropout frequency revealed by the remaining fragments was in all cases low (0 out of 55, 0 out of 93, 1 out of 74 and 3 out of 80 for fragments AL, CD, KL and AG, respectively). It should be noted that although no such estimate can be obtained from amplification results of fragments AB, AH2 and IC given that they are exclusive of a particular arrangement (Fig. 2 and Supplementary Fig. S1), it seems reasonable to assume that their putative dropout frequency is at least as low as that detected for the tested fragments, and in both cases similar to the frequency of cytologically miss-assigned karyotypes that might be incurred even by an experienced researcher. Our amplification results for the test set of 25 males as well as the low dropout frequency inferred from those for the 121 individuals set would support the robustness of the final set of primer pairs and amplification conditions and, therefore, the effectiveness of our strategy to assign karyotypes.

This is to our knowledge the first time that karyotypic frequencies have been estimated for both wild-caught males and females in either *D*. *subobscura* or other Drosophila species. Our study detected a certain deficit of the E_1+2+9+12_ arrangement in females sampled in November 2015, but not in those sampled in November 2014. It is unlikely that this result reflects a possible failure to amplify fragment IC that is exclusive of this arrangement in some females collected in 2015, as it was jointly amplified in males and females in a single 96-well microtiter plate. Even though the frequencies here estimated for the four most common E chromosomal arrangements in the study population (E_1+2+9+12_ included) do not differ significantly between sexes, our observation might deserve further study now made possible by our molecular karyotyping strategy.

Our results using a set of primer pairs and amplification conditions allows the consistent amplification of the set of eight diagnostic fragments and, therefore, the identification of the five E chromosome arrangements under study both in homozygosis and heterozygosis (*i*. *e*., to identify the fifteen possible karyotypes). Moreover, the single fly DNA preparation used yields sufficient DNA not only to amplify these fragments but also to perform a much larger number of amplifications, which would undoubtedly allow double-checking results for the five considered E chromosome arrangements and karyotyping additional simple and complex arrangements of other chromosomes once the corresponding inversion breakpoints were molecularly characterized. Indeed, it would be more than sufficient to identify all the inversions that might segregate in any particular natural population of *D*. *subobscura* (*e*.* g*., between twenty and thirty in different samples collected over a forty-year period at Observatori Fabra^6^), and also in any other population given that the number of all naturally occurring inversions of *D*. *subobscura* is on the order of seventy^22^.

Our time-efficient strategy using DNA extracted from single wild-caught individuals to molecularly karyotype multiple chromosomal arrangements will allow the survey of chromosomal variation in both males and females through time and space. Moreover, the quality and quantity of the DNA extracted from one individual would be sufficient for follow-up experiments such as amplifying and Sanger sequencing some candidate regions in the different chromosomal arrangements of each sampled population (*e*. *g*., inversion breakpoint regions and/or multiple genes within inversions that could be considered candidates for their adaptive character). In the case of inversion breakpoint regions, the use of heterokaryotypes would facilitate the subsequent analysis of variation in each arrangement since in these individuals the allele corresponding to each chromosomal arrangement could be differentially amplified and sequenced. Our strategy also opens up the possibility to study putative epistatic interactions between arrangements affecting the same and/or different chromosomes, interactions that might contribute to the adaptive character of chromosomal polymorphism in Drosophila as well as in other dipteran species. Indeed, this possibility has been so far limited by the large samples that would need to be karyotyped for the analyses to have enough power to detect putative interactions, given the extensive expertise and huge labor required to obtain individual complete karyotypes in very large samples.

## Materials and Methods

### Fly samples and polytene chromosome preparations

A total of 50 and 96 *D*. *subobscura* individuals collected in the outskirts of Barcelona (Observatori Fabra) in November 2014 and November 2015, respectively, were used in the present study to establish a molecular strategy to determine karyotypes for the E chromosome from wild-caught individuals of either sex. Moreover, nine strains homokaryotypic for arrangements E_st_ (*ch cu*, OF1, OF15 and OF28), E_1+2_ (OF21 and OF74), E_1+2+9_ (OF82), E_1+2+9+3_ (FO12B) and E_1+2+9+12_ (OF19) were used to test primer pairs and PCR conditions to amplify diagnostic fragments, and also to establish whether they also worked in heterokaryotypes. Strains OF and FO had been obtained as described in Puerma *et al*.^17^.

Males collected in November 2014 were both cytologically and molecularly karyotyped. For that purpose, they were individually crossed to virgin females of the *ch cu* strain that is homokaryotypic for the A_st_, U_st_, J_st_, E_st_ and O_3+4_ arrangements. These males were frozen upon observing first-instar larvae in the corresponding cultures. Salivary glands of eight third-instar F_1_ larvae of each cross were generally dissected to obtain polytene chromosomes preparations that were used to determine each male karyotype. The probability of correctly assigning a karyotype from eight preparations equals 0.996 under the assumption of additive effects of the different chromosomal arrangements on fitness.

### Molecular procedures

Genomic DNA was extracted from single frozen flies using the Puregen Cell kit B (QIAgen) that yields high-quality DNA in a final 20 μl volume. Primers for PCR amplification were designed from the previously sequenced breakpoint regions^17–19^. PCR reactions were performed either in individual tubes or in 96-well microtiter plates in a total volume of 10 μl using 1 μl of a 1/100 dilution of the high-quality DNA extracted from 1 individual and TaKaRa DNA polymerase (Takara Bio. Inc.).

## Electronic supplementary material

Supplementary Information
