# Supplementary material for: An easy route to the massive karyotyping of complex chromosomal arrangements in Drosophila
Source: Sci Rep. 2017 Oct 5;7:12717. doi: 10.1038/s41598-017-13043-6 (PMC5629216; doi:10.1038/s41598-017-13043-6)
Supplement: Supplementary file 1 — Supplementary Information [file 41598_2017_13043_MOESM1_ESM.pdf]

Supplementary Information

An easy route to the massive karyotyping of complex chromosomal arrangements in Drosophila

Dorcas J. Orengo, Eva Puerma, Unai Cereijo, David Salguero and Montserrat Aguadé*

Departament de Genètica, Microbiologia i Estadística, Facultat de Biologia and Institut de Recerca de la Biodiversitat (IRBio), Universitat de Barcelona, Barcelona, Spain

* Author for Correspondence

Supplementary Table S1. PCR amplification results for 25 females from the 2014 sample. 1

Supplementary Table S2. PCR amplification results for 96 individuals (48 males and 48 females) from the 2015 sample. 2

Supplementary Table S3. Oligonucleotides and PCR conditions for molecular karyotyping. 5

Supplementary Figure S1. Expected PCR results for each chromosomal arrangement. 6

Supplementary Figure S2. Molecular karyotyping of E chromosome arrangements. . . 7

Supplementary Table S1. PCR amplification results for 25 females from the 2014 sample

♀♀	PCR fragment								Molecular Karyotype		AK
	AB	AH2	IC	AL	AG	KL	CD	H1H2			
1H				+	+		+		$E_{\underline{1+2+9}}$	$E_{\underline{1+2+9}}$	+
2H	+		+	+	+	+	+		E_{st}	$E_{\underline{1+2+9+12}}$	+
6H	+					+	+		E_{st}	E_{st}	
7H				+	+		+		$E_{\underline{1+2+9}}$	$E_{\underline{1+2+9}}$	+
10H			+	++	+		+		$E_{\underline{1+2+9}}$	$E_{\underline{1+2+9+12}}$	+
11H	+		+	+	●	+	+		E_{st}	$E_{\underline{1+2+9+12}}$	+
12H	+					+	+		E_{st}	E_{st}	
14H			++	+	+				$E_{\underline{1+2+9+12}}$	$E_{\underline{1+2+9+12}}$	+
15H			+	++	+				$E_{\underline{1+2+9+12}}$	$E_{\underline{1+2+9+12}}$	+
16H	+			+	+	+	+		E_{st}	$E_{\underline{1+2+9}}$	+
17H	+				+	+	+		E_{st}	$E_{\underline{1+2}}$	
18H	+		+	+	+	+	+		E_{st}	$E_{\underline{1+2+9+12}}$	+
19H	+					+	+		E_{st}	E_{st}	
21H	+		+	+	+	+	+		E_{st}	$E_{\underline{1+2+9+12}}$	+
23H				+	+	+	+		$E_{\underline{1+2}}$	$E_{\underline{1+2+9}}$	+
25H	+				+	+	+		E_{st}	$E_{\underline{1+2}}$	
26H	+		+	+	+	+	+		E_{st}	$E_{\underline{1+2+9+12}}$	+
27H	+			+	+	+	+		E_{st}	$E_{\underline{1+2+9}}$	☆
30H	+			+	+	+	+		E_{st}	$E_{\underline{1+2+9}}$	☆
31H		+	+	++	+		+		$E_{\underline{1+2+9+3}}$	$E_{\underline{1+2+9+12}}$	+
50H		+		+	+	+	+		$E_{\underline{1+2}}$	$E_{\underline{1+2+9+3}}$	
51H	+				+	+	+		E_{st}	$E_{\underline{1+2}}$	
52H		+		+	+		+	+	$E_{\underline{1+2+9}}$	$E_{\underline{1+2+9+3}}$	+
53H	+			+	●	+	+		E_{st}	$E_{\underline{1+2+9}}$	★
54H				+	+		+		$E_{\underline{1+2+9}}$	$E_{\underline{1+2+9}}$	+
Total	15	3	9	19	20	17	23				15

+, a single amplification product; ++, two differently sized amplification products. ●, PCR amplification failures of fragments expected to amplify (see Figure S1); ☆, PCR amplification failures detected after the amplification of the AL fragment. The gray background indicates unnecessary amplifications. Total, number of individuals that gave a positive PCR.

Supplementary Table S2. PCR amplification results for 96 individuals (48 males and 48 females) from the 2015 sample

	PCR fragments								Molecular Karyotype		
	AB	AH2	IC	AL	AG	KL	CD	H1H2			AK
A1	+	+		+	+	+	+		E_{st}	$E_{\underline{1+2+9+3}}$	
A2	+			+	+	+	+		E_{st}	$E_{\underline{1+2+9}}$	+
A3	+					+	+		E_{st}	E_{st}	
A4	+					+	+		E_{st}	E_{st}	
A5	+		+	+	+	+	+		E_{st}	$E_{\underline{1+2+9+12}}$	●
A6	+				+	+	+		E_{st}	$E_{\underline{1+2}}$	
A7			+	+	+	+	+		$E_{\underline{1+2}}$	$E_{\underline{1+2+9+12}}$	●
A8			+	++	+		+		$E_{\underline{1+2+9}}$	$E_{\underline{1+2+9+12}}$	+
A9	+				+	+	+		E_{st}	$E_{\underline{1+2}}$	
A10	+		+	+	+	●	+		E_{st}	$E_{\underline{1+2+9+12}}$	+
A11	+					+	+		E_{st}	E_{st}	
A12				+	+	+	+		$E_{\underline{1+2}}$	$E_{\underline{1+2+9}}$	+
B1			+	+	+	+	+		$E_{\underline{1+2}}$	$E_{\underline{1+2+9+12}}$	+
B2	+		+	+	+	+	+		E_{st}	$E_{\underline{1+2+9+12}}$	●
B3	+		+	+	+	+	+		E_{st}	$E_{\underline{1+2+9+12}}$	●
B4	+			+	+	+	+		E_{st}	$E_{\underline{1+2+9}}$	+
B5				+	+		+		$E_{\underline{1+2+9}}$	$E_{\underline{1+2+9}}$	+
B6	+					+	+		E_{st}	E_{st}	
B7	+					+	+		E_{st}	E_{st}	
B8	+		+	+	+	+	+		E_{st}	$E_{\underline{1+2+9+12}}$	+
B9	+					+	+		E_{st}	E_{st}	
B10			+	+	+	+	+		$E_{\underline{1+2}}$	$E_{\underline{1+2+9+12}}$	●
B11			+	+	+		+		$E_{\underline{1+2+9}}$	$E_{\underline{1+2+9+12}}$	+
B12			+	+	+				$E_{\underline{1+2+9+12}}$	$E_{\underline{1+2+9+12}}$	+
C1				+	+	+	+		$E_{\underline{1+2}}$	$E_{\underline{1+2+9}}$	✯
C2	+		+	+	+	+	+		E_{st}	$E_{\underline{1+2+9+12}}$	+
C3			+	+	+				$E_{\underline{1+2+9+12}}$	$E_{\underline{1+2+9+12}}$	+
C4			+	+	+		+		$E_{\underline{1+2+9}}$	$E_{\underline{1+2+9+12}}$	+
C5	+					+	+		E_{st}	E_{st}	
C6	+		+	+	+	+	+		E_{st}	$E_{\underline{1+2+9+12}}$	+
C7			+	+	+		+		$E_{\underline{1+2+9}}$	$E_{\underline{1+2+9+12}}$	+
C8	+				+	+	+		E_{st}	$E_{\underline{1+2}}$	
C9	+				+	+	+		E_{st}	$E_{\underline{1+2}}$	
C10			+	+	+	+	+		$E_{\underline{1+2}}$	$E_{\underline{1+2+9+12}}$	+
C11	+			+	+	+	+		E_{st}	$E_{\underline{1+2+9}}$	+

(continued)

Supplementary Table S2. PCR amplification results for 96 individuals (48 males and 48 females) from the 2015 sample

	PCR fragments								Molecular Karyotype		AK
	AB	AH2	IC	AL	AG	KL	CD	H1H2			
C12			+	++	+				$E_{1+2+9+12}$	$E_{1+2+9+12}$	+
D1	+		+	+	+	+	+		E_{st}	$E_{1+2+9+12}$	+
D2		+		+	+		+	+	E_{1+2+9}	$E_{1+2+9+3}$	+
D3	+			+	+	+	+		E_{st}	E_{1+2+9}	☆
D4	+					+	+		E_{st}	E_{st}	
D5	+		+	+	+	+	+		E_{st}	$E_{1+2+9+12}$	+
D6	+				+	+	+		E_{st}	E_{1+2}	
D7			+	++	+		+		E_{1+2+9}	$E_{1+2+9+12}$	+
D8		+		+	+		+	+	E_{1+2+9}	$E_{1+2+9+3}$	☆
D9			+	++	+				$E_{1+2+9+12}$	$E_{1+2+9+12}$	+
D10			+	+	+		+		E_{1+2+9}	$E_{1+2+9+12}$	+
D11	+				+	+	+		E_{st}	E_{1+2}	
D12			+	+	+	+	+		E_{1+2}	$E_{1+2+9+12}$	+
E1	+					+	+		E_{st}	E_{st}	
E2	+		+	+	+	+	+		E_{st}	$E_{1+2+9+12}$	+
E3	+					+	+		E_{st}	E_{st}	
E4			+	+	+		+		E_{1+2+9}	$E_{1+2+9+12}$	+
E5	+				+	+	+		E_{st}	E_{1+2}	
E6	+				+	+	+		E_{st}	E_{1+2}	
E7	+					+	+		E_{st}	E_{st}	
E8		+		++	+		+	+	E_{1+2+9}	$E_{1+2+9+3}$	☆
E9				+	+	+	+		E_{1+2}	E_{1+2+9}	+
E10	+				+	+	+		E_{st}	E_{1+2}	
E11	+			+	+	+	+		E_{st}	E_{1+2+9}	+
E12				+	+		+		E_{1+2+9}	E_{1+2+9}	+
F1	+					+	+		E_{st}	E_{st}	
F2					+	+	+		E_{1+2}	E_{1+2}	
F3	+		+	+	+	+	+		E_{st}	$E_{1+2+9+12}$	●
F4	+			+	●	+	+		E_{st}	E_{1+2+9}	☆
F5				+	+	+	+		E_{1+2}	E_{1+2+9}	+
F6	+					+	+		E_{st}	E_{st}	
F7	+				+	+	+		E_{st}	E_{1+2}	
F8				+	+	+	+		E_{1+2}	E_{1+2+9}	+
F9	+				+	+	+		E_{st}	E_{1+2}	
F10			+	+	+	+	+		E_{1+2}	$E_{1+2+9+12}$	+

(continued)

Supplementary Table S2. PCR amplification results for 96 individuals (48 males and 48 females) from the 2015 sample

	PCR fragments								Molecular Karyotype		
	AB	AH2	IC	AL	AG	KL	CD	H1H2			AK
F11				++	+		+		E_{1+2+9}	E_{1+2+9}	✯
F12	+				+	+	+		E_{st}	E_{1+2}	
G1	+		+	+	+	+	+		E_{st}	$E_{1+2+9+12}$	+
G2					+	+	+		E_{1+2}	E_{1+2}	
G3	+				+	+	+		E_{st}	E_{1+2}	
G4	+			+	+	+	+		E_{st}	E_{1+2+9}	+
G5	+				+	+	+		E_{st}	E_{1+2}	
G6			+	+	+				$E_{1+2+9+12}$	$E_{1+2+9+12}$	+
G7			+	+	+		+		E_{1+2+9}	$E_{1+2+9+12}$	+
G8		+		+	+	+	+		E_{1+2}	$E_{1+2+9+3}$	
G9			+	+	+		+		E_{1+2+9}	$E_{1+2+9+12}$	+
G10	+					+	+		E_{st}	E_{st}	
G11	+		+	+	+	+	+		E_{st}	$E_{1+2+9+12}$	+
G12	+					+	+		E_{st}	E_{st}	
H1	+					+	+		E_{st}	E_{st}	
H2	+			+	+	+	+		E_{st}	E_{1+2+9}	✯
H3	+			+	+	+	+		E_{st}	E_{1+2+9}	+
H4			+	+	+	+	+		E_{1+2}	$E_{1+2+9+12}$	+
H5	+		+	+	+	+	+		E_{st}	$E_{1+2+9+12}$	●
H6	+					+	+		E_{st}	E_{st}	
H7				+	+	+	+		E_{1+2}	E_{1+2+9}	+
H8	+		+	+	+	+	+		E_{st}	$E_{1+2+9+12}$	+
H9		+	+	++	+		+		$E_{1+2+9+3}$	$E_{1+2+9+12}$	+
H10	+				+	+	+		E_{st}	E_{1+2}	
H11	+			+	+	+	+		E_{st}	E_{1+2+9}	+
H12	+					+	+		E_{st}	E_{st}	
Total	59	6	37	61	77	74	91				45

+, a single amplification product; ++, two differently sized amplification products. ●, PCR amplification failures of fragments expected to amplify (see Figure S1); ✯, PCR amplification failures detected after the amplification of the AL fragment. The gray background indicates unnecessary amplifications. Total, number of individuals that gave a positive PCR.

Supplementary Table S3. Oligonucleotides and PCR conditions for molecular karyotyping.

Frag.	Forward oligonucleotide	Reverse oligonucleotide	size (kb)	T (°C)	Time (min:s)
AB	TCTTGGCTTGGTGCGTTGAC	CTTTTCGTTGCCCGCTAATG	2.3	59	2:00
AH2	CATAGACTTCGTTGATTCCA	GTGTAAACGAGGAGACGAAT	4.7	59	4:30
IC	AGAGGATAGGCATTGAAGTGTCG	GCTGTGGGAAACTATCGGTATGA	3.8	61	3:30
AL	CCTCTGGGCATTCGGACTTC	CGAACACCGAACCCATTTTG	5.7	61	4:00
AG	CATAGACTTCGTTGATTCCA	GTCATTATCGTCACACATTC	2.9	56	2:00
KL	TAGTGACAATACGGGTGATA	ACAACATTAAACTGCGACTC	2.8	59	2:00
CD	TTAACCACCGCAATCACTAT	TTGTTCACCGACGAGAGT	5.5	59	4:00
H1H2	AAGGAGTTGGAAAGCGAAGC	TCCGAGACCCGCAAATCACC	6.3	59	4:00

Frag., Fragment; min, minutes; s, seconds.

Supplementary Figure S1. Expected PCR results for each chromosomal arrangement.

Group	AB			AG							AH2		CD		JD	
Fragment	AB	EF	GH	AG	FB	EH	KL	AK	GAL	H1H2	AH2	KH1	CD	IJ	JD	IC
E_{st}	+	+	+	-	-	-	+	-	-	+	-	-	+	+	-	-
E_{1+2}	-	-	-	+	+	+	+	-	-	+	-	-	+	+	-	-
E_{1+2+9}	-	-	-	+	+	+	-	+	+	+	-	-	+	+	-	-
$E_{1+2+9+3}$	-	-	-	+	+	+	-	-	+	-	+	+	+	+	-	-
$E_{1+2+9+12}$	-	-	-	+	+	+	-	+	+	+	-	-	-	-	+	+
#	1	3	4	8	9	10	7	11	12	5	15	16	2	6	13	14

Sets of redundant fragments are grouped and named by the first fragment considered. #, fragments numbered as in Figure 2; +, positive PCR result; -, negative PCR result. The blue background indicates that these positive PCR amplifications correspond to the long duplication of region A during E_9 inversion process.

Supplementary Figure S2. Molecular karyotyping of E chromosome arrangements

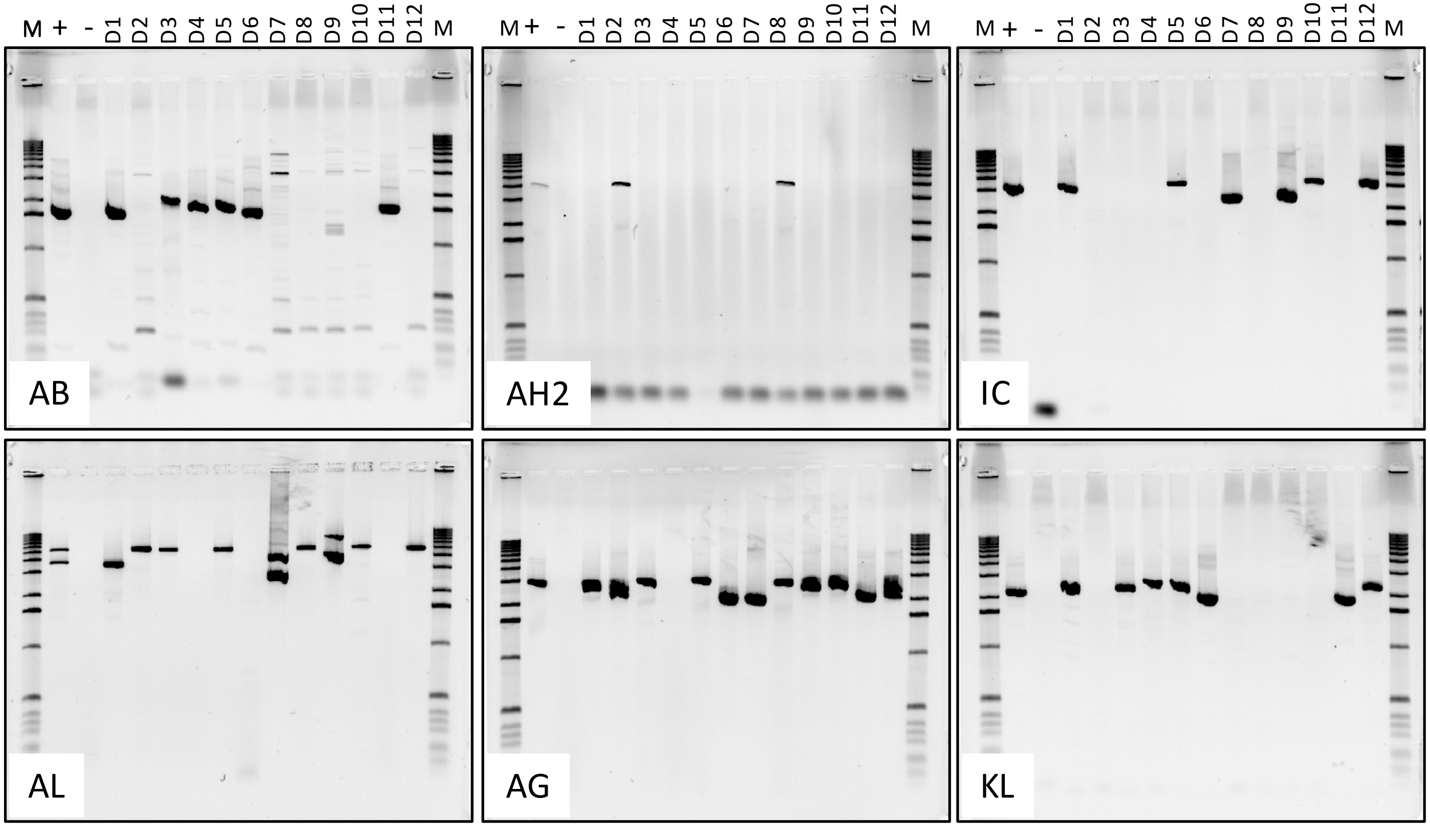

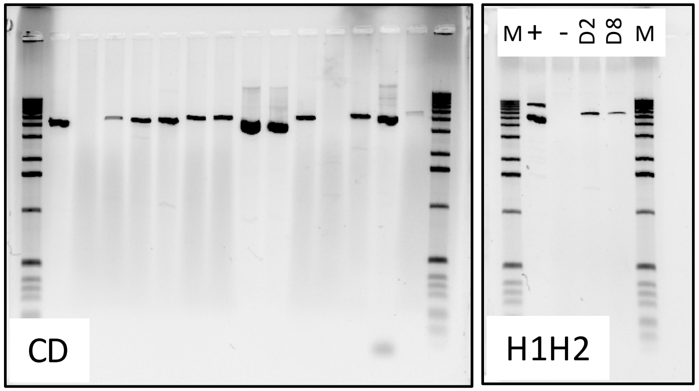

	AB	AH2	IC	AL	AG	KL	CD	H1H2	Inferred Karyotype
D1	+		+	+	+	+	+		$E_{st}/E_{\underline{1+2+9+12}}$
D2		+		+	+		+	+	$E_{\underline{1+2+9}}/E_{\underline{1+2+9+3}}$
D3	+			+	+	+	+		$E_{st}/E_{\underline{1+2+9}}$
D4	+					+	+		E_{st}/E_{st}
D5	+		+	+	+	+	+		$E_{st}/E_{\underline{1+2+9+12}}$
D6	+				+	+	+		$E_{st}/E_{\underline{1+2}}$
D7			+	++	+		+		$E_{\underline{1+2+9}}/E_{\underline{1+2+9+12}}$
D8		+		+	+		+	+	$E_{\underline{1+2+9}}/E_{\underline{1+2+9+3}}$
D9			+	++	+				$E_{\underline{1+2+9+12}}/E_{\underline{1+2+9+12}}$
D10			+	+	+		+		$E_{\underline{1+2+9}}/E_{\underline{1+2+9+12}}$
D11	+				+	+	+		$E_{st}/E_{\underline{1+2}}$
D12			+	+	+	+	+		$E_{\underline{1+2+9}}/E_{\underline{1+2+9+12}}$

Images of the electrophoretically separated PCR products corresponding to each of the eight diagnostic fragments (AB, AH2, IC, AL, AG, KL, CD and H1H2). M, molecular weight markers (1-kb ladder; Life Technologies); +, positive control; -, negative control. Samples in the remaining lanes correspond to 12 of the 48 males collected in November 2015 (Table S3, Supporting information), except for fragment H1H2 with products of only two of these males. The small table inserted in the right lower part gives a summary of the PCR results as well as the inferred karyotype for each individual; +, a single amplification product; ++, two differently sized amplification products.
